# Monitoring patients with uncomplicated common variable immunodeficiency: a systematic review

**DOI:** 10.1186/s13223-022-00661-7

**Published:** 2022-03-09

**Authors:** Erika Yue Lee, Stephen Betschel, Eyal Grunebaum

**Affiliations:** 1 Division of Clinical Immunology and Allergy, Department of Medicine, St. Michael’s Hospital, 30 Bond Street, Toronto, ON M5B 1W8 Canada; 2 Division of Immunology and Allergy, Department of Pediatrics, The Hospital for Sick Children, Toronto, ON Canada; 3 Faculty of Medicine, University of Toronto, Toronto, ON Canada

**Keywords:** Adults, Children, Common Variable Immunodeficiency, CVID, Monitoring, Uncomplicated, Screening

## Abstract

**Background:**

Non-infectious complications have become a major cause of morbidity and mortality in patients with Common Variable Immunodeficiency (CVID). The monitoring of patients with CVID prior to the development of non-infectious complications is not well defined.

**Objective:**

Our objectives were to systematically review the current literature on the monitoring of CVID patients without non-infectious complications and to develop recommendations for such monitoring.

**Methods:**

MEDLINE and EMBASE were searched from January 1^st^, 2000 to March 25^th^, 2021. Studies on any aspects of CVID monitoring were included. Studies that included only children, on monitoring CVID patients with existing non-infectious complications, or in the format of case reports were excluded.

**Results:**

Nine studies on CVID monitoring, including 3 cohort studies, 3 experts’ opinions, 2 consensus statements and a single guideline report were identified. These studies revealed that clinical assessment and bloodwork were preformed every 6 to 12 months in asymptomatic patients. Some centers performed computerized tomography scan of the chest every 2–5 years to identify chronic lung disease, although the majority did chest imaging in accordance with clinical indications. Pulmonary function tests were done annually at most centers. Most studies did not address the role of abdominal imaging to screen for liver diseases or endoscopy to screen for gastric cancer in asymptomatic patients with uncomplicated CVID.

**Conclusions:**

There is paucity of evidence-based information to guide the routine monitoring of CVID patients without non-infectious complications. Prospective studies are needed to determine the best monitoring practices in this group of patients.

## Introduction

Common variable immunodeficiency (CVID) is characterized by impaired production of immunoglobulins. Its prevalence is estimated at 1:25,000 to 1:50,000, and is the most common human symptomatic inborn error of immunity [1]. Affected patients often present with recurrent infections, although more recently many are also identified with autoimmunity and/or lymphoproliferation [2]. The majority of patients with CVID are diagnosed at 20–40 years of age, although 20%-30% might be diagnosed earlier [1, 3]. Typical laboratory features include reduced blood immunoglobulins (Ig) and impaired production of specific antibody to vaccinations or native infections. Because of the disease heterogeneity, several groups have proposed criteria for diagnosing CVID [2, 4–6]. An example of the original diagnostic criteria proposed in 1999 was listed in Box 1 and provided the framework for the other revised or modified criteria [4].

The clinical spectrum of CVID is diverse but can be grouped into two main phenotypes. The first and largest group includes patients with CVID who suffer predominantly from recurrent infections [7, 8]; it can be referred to as CVID with infections only or uncomplicated CVID. The second group of patients initially present with or subsequently develop non-infectious manifestations and can be referred to as CVID with non-infectious complications. These complications can be categorized into autoimmune cytopenia, unexplained enteropathy, lymphoproliferation and others. They can manifest as progressive lung derangement, hematological and non-hematological autoimmunity, inflammatory bowel and liver diseases, lymphoid hyperplasia, and malignancies, as detailed in Box 2 [1, 7–9].

The introduction of life-long intravenous immunoglobulin (IVIg) or subcutaneous immunoglobulin (SCIg) replacement therapy for patients with CVID has led to a marked decrease in the incidence of infections, the rate of hospitalization, and death from acute bacterial infections [1, 10–12]. The reduction of infectious complications has led to an even greater appreciation of the morbidity and mortality associated with the non-infectious complications. Indeed, patients in the second group have an estimated 11 times higher risk of death compared to those with CVID without non-infectious complications [13].

The considerable impact of the non-infectious complications emphasizes the importance of their timely detection, especially among patients with CVID that have been free of them. Early identification of non-infectious complications is possible through diverse laboratory tests, imaging, and procedures. However, the precise frequency and extent of surveillance of such interventions in asymptomatic patients with uncomplicated CVID is not well defined. Moreover, such evaluations might pose unnecessary and possibly dangerous burden on the patients as well as extensive demands on the health care system. Therefore, wise use of the limited resources is prudent.

Accordingly, we performed a systematic literature review on the monitoring of CVID patients without non-infectious complications. We focused our efforts on the most clinically relevant and readily available monitoring tools including clinical assessment, laboratory investigations, imaging, and pulmonary function tests (PFT). Importantly, we identified major gaps in the evidence supporting the monitoring of patients with CVID for non-infectious complications.

Box 1. The original ESID/PAGID diagnostic criteria for Probable CVID proposed in 1999

**Table:** 

1. Marked decrease in IgG (at least 2 SD below the mean for age) and in at least one of the isotypes, IgM or IgA, AND	
2. All of the following criteria:	
a. Onset of immunodeficiency greater than 2 years of age	
b. Absent of isohemagglutinins and/or poor response to vaccines	
c. Secondary causes of hypogammaglobulinemia have been ruled out	

### Box 2. Complications classified based on the CVID clinical phenotypes [1, 7–9]

**Table:** 

Clinical phenotypes	Common manifestations	Possible evaluation
I. Infection only	Sinusitis, otitis Pneumonia, empyema Diarrhea Arthritis	CBC Cultures: sputum, nasal swab, stool, joint fluid X-ray or CT of sinus, chest
IIa. Non-infectious complication: *Autoimmune cytopenia*	ITP AIHA Neutropenia	CBC, blood film Bone marrow aspirate and biopsy to rule out malignancy
IIb. Non-infectious complication: *Unexplained enteropathy*	Malabsorption Chronic diarrhea	Iron study, vitamin B12 Celiac screen Stool cultures Endoscopy
IIc. Non-infectious complication: *Polyclonal lymphoproliferation*	LIP LAD Granulomatous disease	Bronchoscopy ± biopsy Biopsy of LAD or granuloma
IId. Non-infectious complication: *Others*^a^ (not included in Chapel’s classification)	Bronchiectasis Hepatomegaly NRH Splenomegaly Other autoimmunity Malignancy	Chest imaging Pulmonary function test Abdominal ultrasound Biopsy of spleen, liver

^a^For the purpose of monitoring, *Others* refer to the non-infectious complications that are also part of CVID monitoring and thus included in this table

ITP immune thrombocytopenic purpura, AIHA autoimmune hemolytic anemia, LIP lymphoid interstitial pneumonitis, LAD lymphadenopathy, NRH nodular regenerative hyperplasia

## Methods

### Search strategy

We conducted this systematic review according to the Preferred Reporting Items for Systematic Reviews and Meta-Analyses (PRISMA) statement. The following electronic databases were used for a comprehensive literature search: MEDLINE, EMBASE, PubMed, The Cochrane Library and clinicaltrials.gov.

The search terms used in MEDLINE and EMBASE were Common Variable Immunodeficiency AND (consensus or practice guideline), as well as Common Variable Immunodeficiency AND (monitor or follow up or screen) AND each of the following terms separately: (bloodwork or laboratory testing), (imaging or radiography or x-ray or CT or ultrasound), (pulmonary function test or spirometry). These search terms were also used in the rest of databases. Studies published since 2000 in English language and in humans were retrieved. Age group was limited to all adults (19 plus years). The search of databases was performed between January 25th, 2020 and March 25th, 2021.

### Study selection and data extraction

**Fig. 1:**
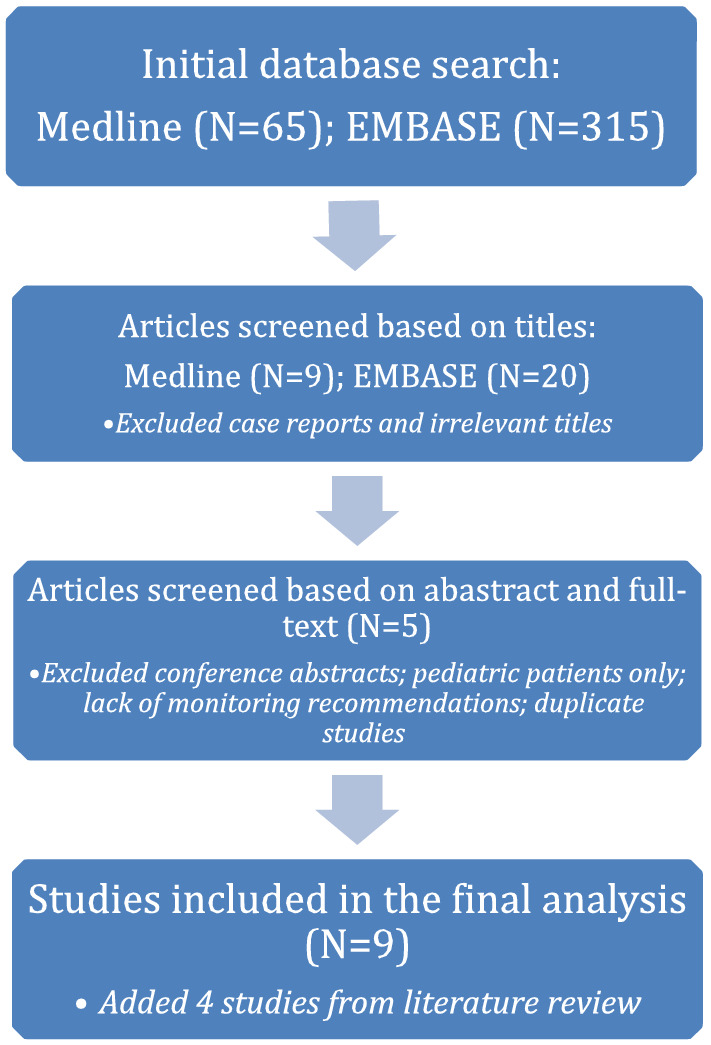
Flowchart showing search strategies and selection of studies

**Fig. 2:**
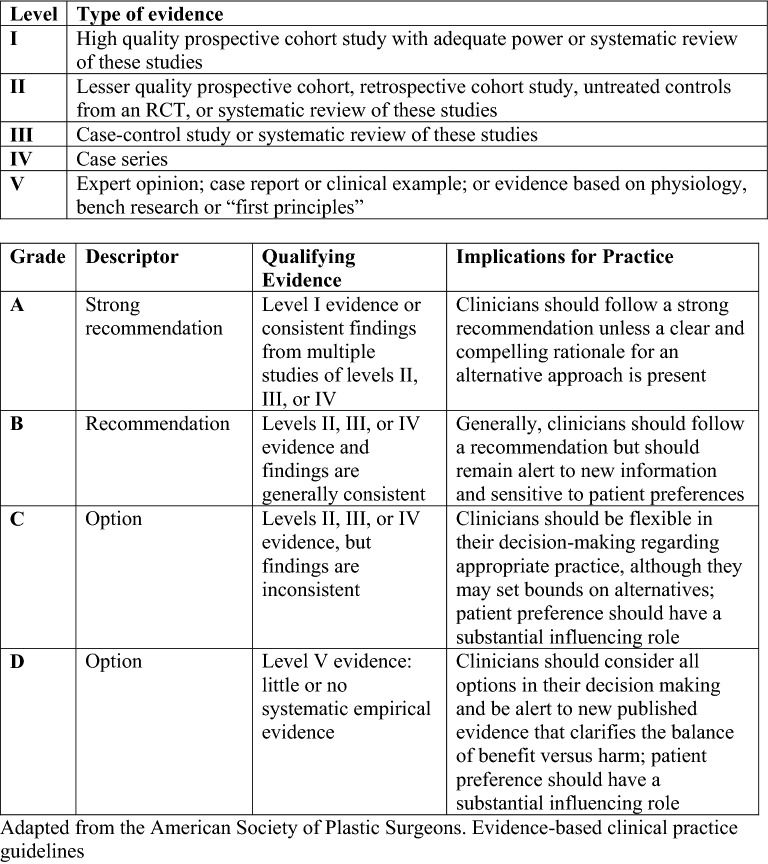
Levels of Evidence for Prognostic Studies (top) and Grade Practice Recommendations (bottom) [14]

The results of the search and selection of studies were summarized in Fig. 1. Our initial literature search identified 65 articles in MEDLINE and 315 articles in EMBASE. We screened titles followed by abstracts and full texts for the studies that included recommendations of monitoring. After exclusion of case reports, exclusively pediatric and repeated studies, 5 peer-reviewed articles were identified and included in the analysis. An additional 4 studies were identified through literature review resulting in 9 studies that were included in the final analysis. Data extraction from these articles included study setting, extent (including modality) and frequency of monitoring. We assessed the levels of evidence and grade of recommendations for each article as described in Fig. 2 [14].

### Risk of bias assessment

Two reviewers independently performed the quality assessment and assessed the risk of bias of the included studies. The Cochrane risk of bias tool was used. Any difference in opinion was resolved through discussion between the reviewers.

## Results

We identified 9 papers that provided recommendations or guidance on monitoring patients with uncomplicated CVID. These papers are a mix of cohort studies (3 papers) [15, 16, 22], experts’ opinions based on their clinical experience (3 papers) [11, 17, 19], consensus statements (2 papers) [2, 20], and one guideline [21]. Although two of the cohort studies were from the same centre, both were included in this review because they provided specific recommendations on routine monitoring [15, 16]. We did not include a survey study on monitoring patients with primary immunodeficiency because the frequency of monitoring and testing were not specified in the study [18]. We also shared our experience with CVID monitoring at our centre. These results are summarized in Table 1. Overall, there is a lack of a uniform practice for monitoring patients with uncomplicated CVID. There is also no uniform consensus on the frequency of imaging and pulmonary function tests to screen for non-infectious complications.

**Table 1:** Frequency and modality of monitoring in patients with uncomplicated CVID by studies

No	Study and Level of Evidence	Clinical assessment	Laboratory testing	Chest imaging	Abdominal imaging	PFT
1	Quinti et al. [11] Expert’s opinion Level V. Grade D	Not mentioned	Every 3 months: Ig, CBC, lymphocyte subsets, chemistries, culture tests	Every 4 years: CT chest and sinus	Every 1 year: AUS Every 2 years: upper endoscopy	Not mentioned
2	Cunningham-Rundles [19] Expert’s opinion Level V. Grade D	Every 12 months	Every 6–12 months: Ig Every 12 months: CBC, chemistry, albumin, creatinine, liver enzymes	Baseline then as needed: HRCT is preferred	Not mentioned	Every 1 year
3	Abolhassani et al. [17] Expert’s opinion Level V. Grade D	Every 3–6 months	Every 3–6 months: hematologic testing Every 12 months: TSH Regular check: HCV PCR	As needed	Every 1 year: AUS Every 2 years: upper ± lower endoscopy	Every 1–2 years
4	Maarschalk-Ellerbroek et al. [15] Cross-sectional cohort study (N = 47) Level II. Grade B	Not mentioned	Every 6–12 months: Ig	Baseline: CT	Not mentioned	Baseline
5	Buckley [21] Guideline Level V. Grade D	Not mentioned	Every 6–12 months: Ig, creatinine, liver enzymes Every 12 months: HCV PCR	Baseline: CT	Not mentioned	Every 1 year
6	Bonilla et al. [2] Consensus Level V. Grade D	Scheduled follow-ups (frequency not specified)	Every 6-12 months: liver enzymes Regular check: Ig, CBC, creatinine, urea (frequency not specified)	Baseline: HCRT	Not mentioned	Every 1 year
7	Caliskaner et al. [23] Retrospective cohort study (N = 25) Level II. Grade B	Every 3–4 weeks	Every 3–4 weeks: CBC Every 3 months: Ig, lytes, urea, creatinine; urinalysis; stool O&P Every 6 months: total protein, albumin, glucose, LDH, liver enzymes; C3, C4; ANA, dsDNA, thyroid autoantibodies Every 12 months: TSH, T4, T3; CEA, AFP, CA19-9	Every 2 years: HRCT	Every 2 years: AUS	Every 6 months
8	Janssen et al. [16] Prospective cohort study (N = 55) Level II. Grade B	Not mentioned	Not mentioned	Every 5 years: CT	Not mentioned	Baseline then as needed
9	Bethune et al. [20] Consensus Level V. Grade D	Every 6 month: weight; every 12 month: LN and abdomen exams	Every 6 months: Ig, CBC, liver enzymes	Baseline: HRCT Every 5 years: HRCT (if ongoing respiratory tract infections^)	Every 1 year: AUS (no consensus)	Every 1–3 years (no consensus)
10	Our centre Expert’s opinion Level V. Grade D	Every 1 month	Every 6 months: Ig, CBC, LDH, albumin, creatinine, liver enzymes; urinalysis	Baseline: CT chest As needed: CXR or CT chest	Every 1 year: AUS	Every 1 year
11	Summary of suggested frequency and type of monitoring	Every 1–12 months	Every 6–12 months: Ig, CBC, creatinine, liver enzymes	Baseline: CT Every 2–5 years or as needed: CT or CXR	Every 1–2 years: AUS, endoscopy (expert’s opinion)	Every 1–3 years

*LN* lymph node, *Ig* immunoglobulin, *CXR* chest X-ray, *HRCT* high-resolution CT, *AUS* abdominal ultrasound

^ to monitor for bronchiectasis

### A. Clinical assessment

Lifelong follow-up by physicians with expertise in CVID should be part of the routine monitoring [7]. However, there are no studies on the optimal frequency of clinical monitoring. A single expert opinion recommended detailed clinical assessment every 12 months in stable patients, which should be increased to every 3 to 6 months or sooner in patients with comorbidities [19, 20]. Similarly, a consensus guideline suggested monitoring including abdomen and lymph node examination every 6–12 months, with weight follow-up every 6 months. At our academic center that also serves as a training site for residents, patients receiving IVIg at our site are seen by a physician at least monthly. This approach provides longitudinal care to patients with more complex CVID, enables familiarity of this condition and enhances competency of trainees. Patients receiving IVIg at different infusion sites or SCIg at their homes are followed every 6–12 months at our center either in person or via telemedicine, relying on examinations performed by local medical teams.

### B. Laboratory testing

Bloodwork enables monitoring of Ig levels, hematopoietic lineages, liver function and for possible lymphoproliferative disorders. Initial evaluation and diagnosis of CVID include measurement of Ig isotypes, antibody titers to vaccines and natural infections, complete blood count (CBC), liver enzymes, creatinine, and PCR testing for HAV, HBV, HCV and HIV [20, 21]. Analysis of peripheral lymphocyte subsets by flow cytometry is usually added [11]. In recent years, additional flow cytometric analysis of B-cell subgroup, if available, is added to better characterize the immunophenotype of CVID [2, 22]. Most studies, consensus guidelines, and the practice at our center are to repeat bloodwork in patients with uncomplicated CVID every 6–12 months [19–21]. A single expert opinion suggests bloodwork every 3 months [23]. While all recommend measuring Ig, some also measure CBC and liver enzymes, as well as kidney function and blood-borne viral infections. At our center, we also measure LDH. Notably, additional bloodwork can be guided by clinical assessment and/or previous abnormal results. For example, at our center, Ig levels are repeated more frequently if patients have an increased number of infections, experience a significant change in weight, change the Ig product, or there is a change in the route of Ig administration. At our center, urinalysis and urine culture are monitored every 6 months, due to the risk of asymptomatic ureaplasma urinary tract infection [24].

### C. Imaging

Diagnostic imaging may help screen for chronic lung disease, lymphoproliferation and liver diseases. However, the frequency and extent of monitoring as well as the specific modality remain highly variable. To screen for chronic lung diseases in CVID, CT chest is considered the gold standard for detecting initial bronchial changes [25]. For baseline imaging, some advocate high-resolution CT (HRCT) chest [18–20], whereas others use regular CT chest [15, 16], which is also the preference at our center. Most recommend repeat CT scan every 2–5 years, depending on the frequency of respiratory infections or stratification to risk groups. At our center, chest X-ray is used primarily for patients with CVID without preexisting lung disease who develop acute respiratory symptoms. A single expert opinion also suggests CT sinus as routine screening [11].

On the other hand, the recommendation on the use of abdominal imaging to monitor patients with uncomplicated CVID is limited. Only 3 of the 9 evaluated papers mention the use of abdominal imaging to screen for liver diseases, abdominal lymphadenopathy and splenomegaly [11, 20, 23]. Two experts’ opinions suggest yearly abdominal ultrasound (AUS) [11, 17], which is also practiced at our center. AUS is of particular importance for patients with unexplained elevated liver enzymes [26]. The frequency of abdominal imaging ranges from every 1 to 2 years to as needed.

Lastly, the recommendation on the role of upper and/or lower endoscopy to screen for gastrointestinal (GI) cancer is very limited. One expert’s opinion suggests routine endoscopy every 2 years as part of the uncomplicated CVID monitoring, or sooner when indicated [11]. Another expert’s opinion suggests upper and/or lower endoscopy every 2 years, especially when GI complications are suspected [17]. The role of routine endoscopy in uncomplicated CVID monitoring is not mentioned in the remaining 7 papers evaluated in this review.

### D. Pulmonary function test

Pulmonary function test (PFT) is useful for monitoring obstructive and restrictive lung diseases. Although it is easy to perform and does not involve exposure to irradiation, it is not adequate in assessing for parenchymal lung diseases. While most agree that the common occurrence of bronchiectasis in CVID justifies performing PFT upon diagnosis, the frequency that this test should be repeated is not clear [2, 16, 18]. Some suggest repeating PFT every 1 year [19] to every 3 years [20], while others recommend PFT as needed [16]. At our center, PFT are repeated annually, or sooner based on clinical assessment and/or radiographic changes.

## Discussion

**Fig. 3:**
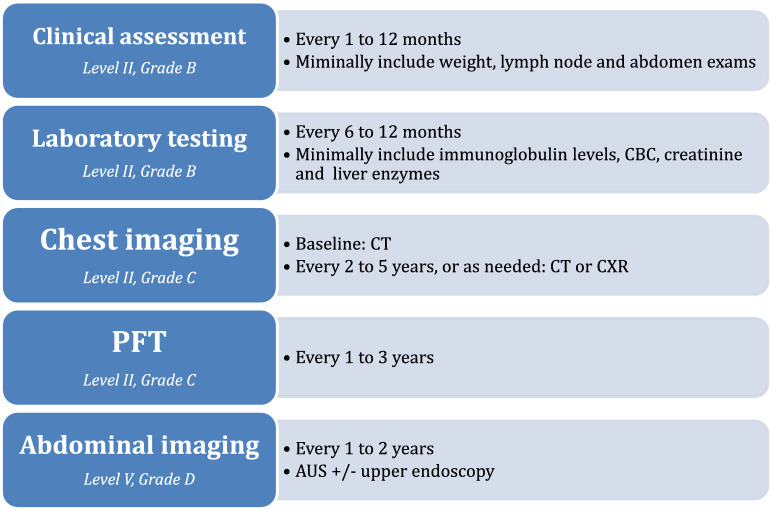
Recommendations on monitoring patients with uncomplicated CVID

Overall, there are very few studies on long-term monitoring of patients with uncomplicated CVID. Importantly, our systematic review identified various practices in monitoring this group of patients while receiving Ig replacement therapy. Until further evidence becomes available, it is standard to perform clinical assessment and bloodwork every 6–12 months in stable patients; it is common to perform PFT annually and chest imaging as needed to screen for lung diseases; it is infrequent to perform abdominal imaging to screen for liver diseases and lymphoproliferation; and it is rare to perform routine endoscopy to screen for gastric cancer. Other than needing regular scheduled follow-ups with physicians experienced in CVID and periodic monitoring of Ig levels, the practice of routine imaging and PFT differs across centers. We developed the final recommendations of monitoring patients with uncomplicated CVID based on very limited evidence and mostly expert’s opinions and summarized the recommendations in Fig. 3.

CVID is a chronic immunologic disorder that requires lifelong monitoring. The goals of CVID management include preventing recurrent infections, as well as screening, monitoring, and treating complications associated with CVID. The clinical practice to achieve the former goal is well delineated and includes Ig replacement and/or antibiotic prophylaxis [13, 27–29]. The practice to achieve the latter goals is variable due to a lack of consensus on CVID monitoring, especially in patients without non-infectious complications. Routine clinical assessment ranges from every 1 to 12 months, depending on the comorbidities. Investigations can be divided into baseline, routine and as needed. Baseline testing is similar across centers and as-needed testing is guided by clinical assessment and patients’ comorbidities. However, the practice of routine testing to screen for non-infectious complications in CVID such as chronic lung diseases, liver diseases or gastric cancer differs across centers.

Common chronic lung diseases seen in CVID patients include interstitial lung disease (ILD) that ranges from LIP to granulomatous lung disease and that is sometimes considered disease-intrinsic [7, 8]. They also include airway disease (AD) like bronchiectasis due to recurrent pulmonary infections and that is likely considered disease-associated [9, 30, 31]. While it is acknowledged that CVID patients with known lung diseases need to be closely monitored [31], our review identified studies that support the need to screen for lung diseases in asymptomatic patients with uncomplicated CVID. For example, one study showed that silent progression to ILD or AD could occur in the absence of any respiratory infections and despite receiving optimal medical treatment [16]. The study underscored the need for routine chest imaging to screen for asymptomatic ILD or AD. Hence, regular chest imaging should be included in monitoring uncomplicated CVID, as it may allow early detection and prompt intervention to potentially prevent progression to irreversible lung damages [15, 16]. What remains unclear is the modality and frequency of routine imaging in CVID patients without any known lung complications. One reason could be that physicians try to minimize radiation exposure in patients with CVID due to the higher baseline risk for malignancies as well as the possibility of increased cellular radio-sensitivity [11, 32, 33].

Further, common liver diseases seen in CVID patients include granulomatous infiltration, NRH and cirrhosis with portal hypertension (PHTN) [9, 26, 34]. A recent cohort study showed a higher mortality rate in patients with CVID complicated by liver diseases, especially if there was also concomitant cirrhosis and/or PHTN [26]. The study suggests that patients should be screened regularly for early detection of liver disease and monitored for any progression using liver enzymes and abdominal ultrasound (AUS). A separate study showed that on annual AUS, CVID patients developed progressive hepatic and/or splenic enlargement as well as radiographic changes in liver texture from smooth to coarse that preceded any biochemical changes [35]. The study suggests that routine AUS is necessary to detect these damages early but does not show if identifying these changes early translates into a better outcome. Overall, there are very few studies on the role of abdominal imaging for monitoring liver diseases in CVID patients. As such, it is not part of CVID monitoring in any of the consensus statements or existing guidelines. Nonetheless liver diseases can be asymptomatic in the initial stage, so early detection ensures more regular monitoring and allows prompt referral to hepatology service when necessary. However, most available treatment for liver diseases provides only symptomatic relief but is not curative, so it remains unknown if the early detection and/or intervention will lead to an improved survival.

In addition to liver diseases, several other gastrointestinal (GI) diseases are also known to be associated with CVID. Examples include inflammatory bowel disease, CVID-related enteropathy, intestinal lymphangiectasia and non-specific malabsorption [17]. Usually, patients with these GI diseases are symptomatic, which would prompt physicians to order investigations such as upper and/or lower endoscopy. On the other hand, patients with CVID are also at an increased risk for gastric cancer and likely asymptomatic in the pre-malignant or early stage [11, 17, 36]. Yet, there are no consensus guidelines that address the role of routine endoscopy to screen for gastric cancer in asymptomatic CVID patients. One study showed that more than 1/3 of CVID patients had at least one pre-malignant and/or malignant GI lesions during routine upper endoscopy; most of these patients did not have any active GI symptoms and thus would not be eligible for endoscopy if testing were ordered based on symptoms alone [36]. However, gastric cancer was shown to be the leading cause of death in a cohort of Italian patients with CVID, and early diagnosis was associated with a longer survival time [37]. While the use of routine endoscopy in monitoring asymptomatic CVID patients remains unclear, physicians should have a lower threshold of ordering an endoscopy in patients with any GI complaints in this at-risk cohort.

Given the substantial morbidity and mortality in CVID patients, screening for non-infectious complications remains a priority during routine monitoring. Early detection of these complications allows prompt referrals to appropriate specialists but also a coordinated approach using multidisciplinary care. In fact, the framework of multidisciplinary care is often adopted in complex and/or chronic diseases and has been shown to improve outcomes [38, 39]. Since CVID can become a complex and chronic disease with multi-system manifestations, there is a demand for interdisciplinary collaboration among physicians with expertise in infectious disease, respirology, hematology, gastroenterology, rheumatology, otolaryngology and so on [40]. Ultimately, early detection of complications through routine monitoring allows early adoption of a multidisciplinary approach to address the diverse needs and develop highly specialized management plans, which can potentially improve outcomes in patients with uncomplicated CVID [41].

This systemic review identified several areas in routine CVID monitoring that warrant more studies in the near future. First, liver disease is common in CVID and associated with a poor prognosis [26], so it would be worthwhile to study the utility of AUS as a screening tool during routine monitoring. One may study the clinical significance of radiographic changes seen on routine AUS. One may also study if AUS should be done irrespective of liver enzymes. The findings of these potential studies will contribute to the understanding in the role of early detection and/or intervention in liver diseases and preventing morbidity and mortality. Second, despite an increased risk of malignancy in patients with CVID, the current screening practice is the same as for the general population. More studies are needed to evaluate when and how to screen for cancer, especially lymphoma and gastric cancer, in addition to clinical assessment, routine bloodwork and standard cancer screening protocols. Third, the natural history of progression to non-infectious complications in CVID patients, other than GLILD, is mostly unknown. Prospective studies on the development of non-infectious complications like liver diseases can shed light to enable physicians to better understand the disease course. Accumulating more studies in CVID monitoring would generate more evidence for creating guidelines, which would then set the standard of practice regionally and internationally.

A few limitations of our findings merit consideration. The first limitation is the lack of high-quality studies being included in this systematic review. Conducting meaningful randomized controlled studies is exceedingly difficult in uncommon diseases like CVID. As such, the paucity of studies on CVID monitoring results in the lack of evidence-based guidelines and uniform recommendations. The second limitation is that our findings in this review are limited to current practice in monitoring patients without CVID complications. One may argue that monitoring CVID patients with pre-existing autoimmune cytopenia is likely similar other than needing more frequent bloodwork and co-managing with hematologists. However, monitoring CVID patients with other non-infectious complications, especially chronic lung diseases, is more complex and would warrant a separate review. The third limitation is that our findings are based on CVID patients who receive regular Ig replacement therapy. In patients who meet the diagnostic criteria for CVID but do not have recurrent infections, the decision of when to initiate Ig replacement therapy remains controversial [28]. Although there is no evidence that Ig treatment alters the disease course of non-infectious complications in CVID patients, it is unclear if the monitoring practice in uncomplicated CVID patients on Ig treatment would be the same as in the ones not on Ig treatment.

## Conclusion

Our review shows that there is very limited information on how to best monitor patients with CVID prior to the development of non-infectious complications. In stable patients, current recommendations consistently support clinical assessment and bloodwork at least every 12 months. Most expert’s opinions recommend PFT every 1 to 3 years while the practice of routine chest imaging is inconsistent. The benefits of annual abdominal imaging to screen for liver diseases and endoscopy to screen for gastric cancer need to be further studied. Developing a uniform practice for monitoring patients with uncomplicated CVID will allow more efficient and effective care, as well as optimize healthcare resource utilization in the era of “Choosing Wisely”.

## Data Availability

Not applicable.

## Abbreviations

CVID: Common variable immunodeficiency
IVIG: Intravenous immunoglobulin replacement
SCIG: Subcutaneous immunoglobulin replacement
CXR: Chest X-Ray
PFT: Pulmonary function test
AUS: Abdominal ultrasound
PHTN: Portal hypertension
NRH: Nodular regenerative hyperplasia

## Code availability

Not available.
